# Supplementary figures and images for: Skin Wound Healing: The Impact of Treatment with Antimicrobial Nanoparticles and Mesenchymal Stem Cells
Source: J Xenobiot. 2025 Jul 18;15(4):119. doi: 10.3390/jox15040119 (PMC12285939; doi:10.3390/jox15040119)

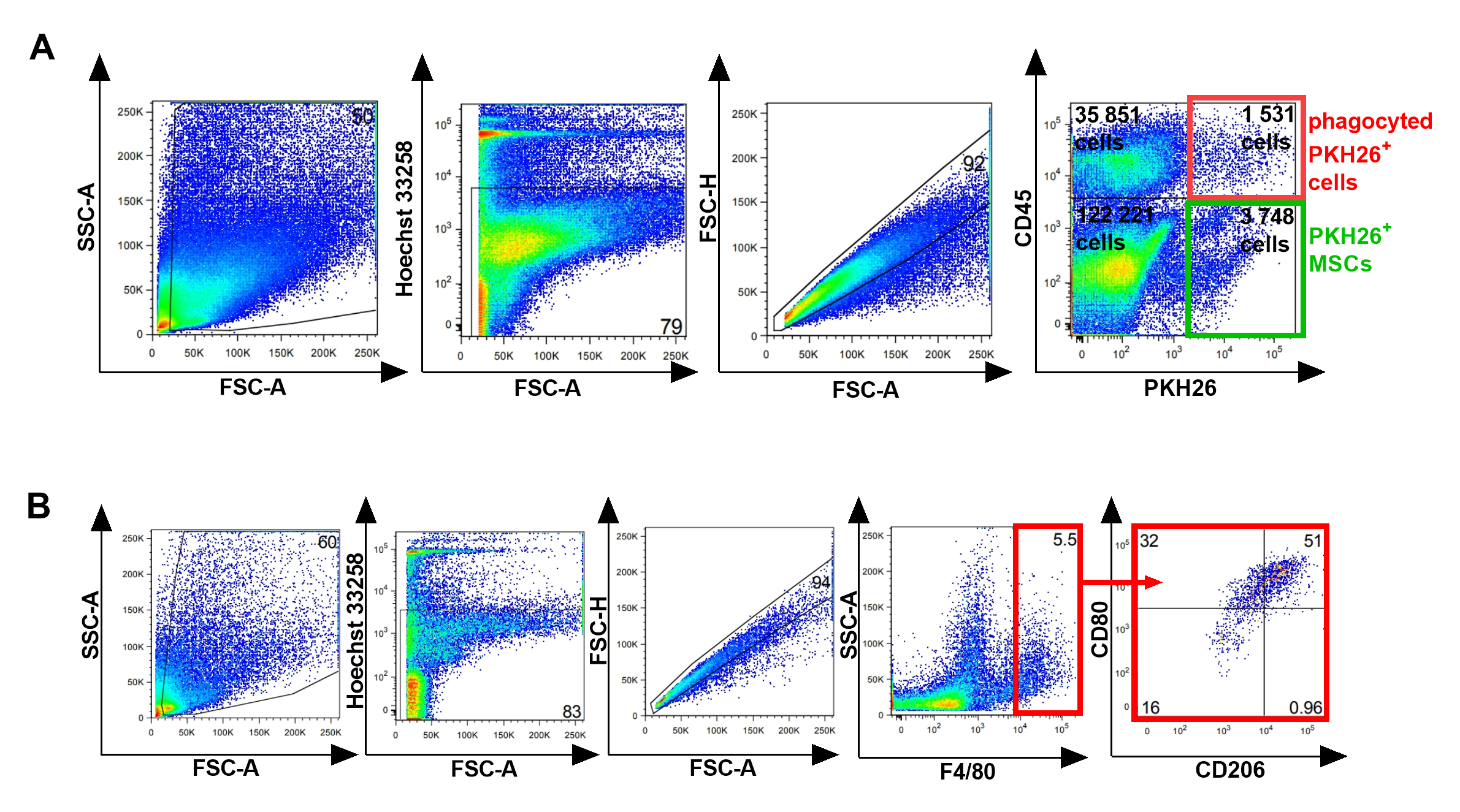

Supplement: Supplementary file 1 [file jox-15-00119-s001.zip › Supplementary Figure S2.tif]

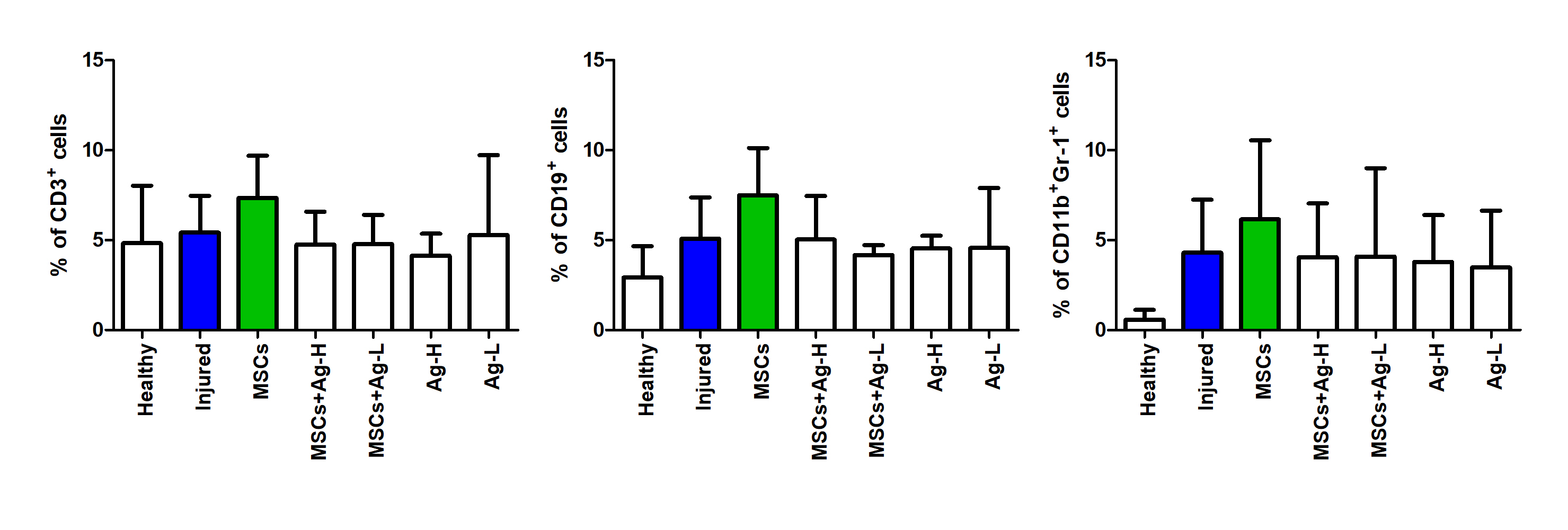

Supplement: Supplementary file 1 [file jox-15-00119-s001.zip › Supplementary Figure S3.jpg]

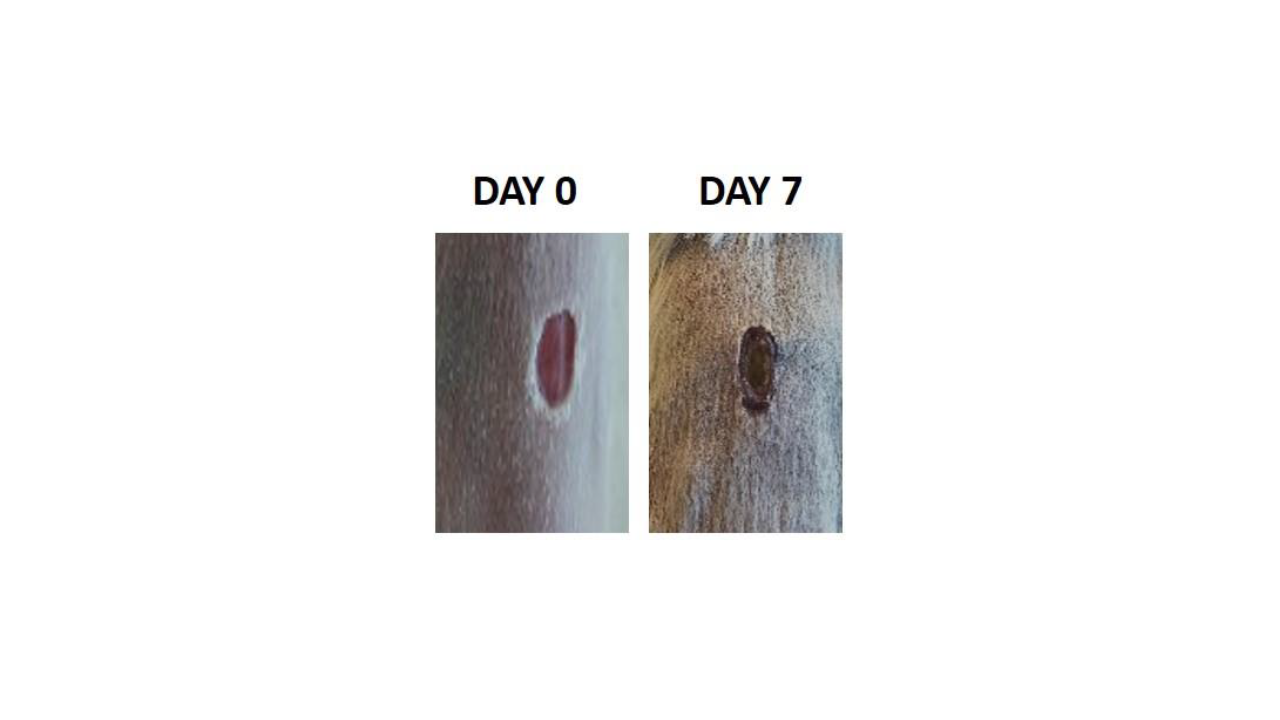

Supplement: Supplementary file 1 [file jox-15-00119-s001.zip › Supplementary Figure S4.tif]

**Supplementary Figure 1.** A representative photograph of a mice treated with MSCs/NP.

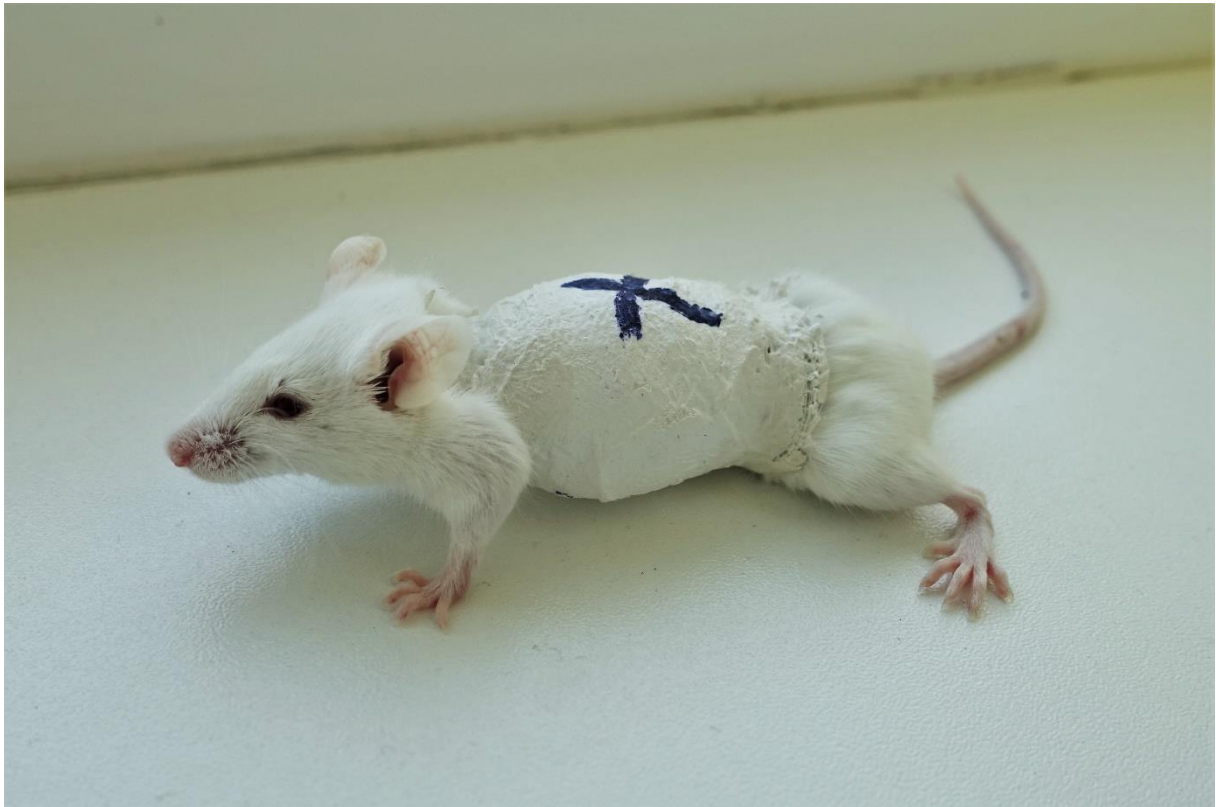

Supplement: Supplementary file 1 [file jox-15-00119-s001.zip › Supplementary Figure S1.pdf]
